# Re-Evaluation Method by Index Finger Position in the Face Area Using Face Part Position Criterion for Sign Language Recognition

**DOI:** 10.3390/s23094321

**Published:** 2023-04-27

**Authors:** Noriaki Hori, Masahito Yamamoto

**Affiliations:** 1 Graduate School of Information Science and Technology, Hokkaido University, N-14, W-9, Kita-ku, Sapporo 060-0814, Japan; 2 Center for Human Nature, Artificial Intelligence, and Neuroscience, Hokkaido University, N-12, W-7, Kita-ku, Sapporo 060-0812, Japan

**Keywords:** sign language recognition, AUTSL, SAM-SLR, MMPose, OpenPose

## Abstract

Several researchers have proposed systems with high recognition rates for sign language recognition. Recently, there has also been an increase in research that uses multiple recognition methods and further fuses their results to improve recognition rates. The most recent of these studies, skeleton aware multi-modal SLR (SAM-SLR), achieved a recognition rate of 98.00% on the RGB video of the Turkish Sign Language dataset AUTSL. We investigated the unrecognized parts of this dataset and found that some signs where the fingers touch parts of the face were not correctly recognized. The proposed method is as follows: First, those with slight differences in top-1 and top-2 evaluation values in the SAM-SLR recognition results are extracted and re-evaluated. Then, we created heatmaps of the coordinates of the index finger in one-handed sign language in the face region of the recognition result in the top-1 to top-3 training data of the candidates based on the face part criteria, respectively. In addition, we extracted four index finger positions from the test data where the index finger stayed longer and obtained the product of the heatmap values of these positions. The highest value among them was used as the result of the re-evaluation. Finally, three evaluation methods were used: the absolute and relative evaluation with two heatmaps and an evaluation method integrating the absolute and relative evaluation results. As a result of applying the proposed method to the SAM-SLR and the previously proposed model, respectively, the best method achieved 98.24% for the highest recognition rate, an improvement of 0.30 points.

## 1. Introduction

It is said that there are 70 million people in the world who use sign language. According to official World Organization statistics for 2022 (http://www.who.int/news-room/fact-sheets/detail/deafness-and-hearing-loss accessed on 9 April 2023), approximately 430 million people (more than 5% of the world’s population) will need rehabilitation to address their disabling hearing loss. It is also estimated that by 2050, nearly 2.5 billion people will have some degree of hearing loss, and at least 700 million will require hearing rehabilitation. Sign language recognition research has two main categories: isolated sign language and continuous sign language. The former are datasets of sign language videos ranging from 100 to 2000 words. Currently, the recognition rate for a vocabulary of 200 words exceeds 98%, which is getting close to the level of human recognition. We are working on the former and believe that increasing the recognition rate of isolated signs can be used in future studies of continuous signs. In the field of sign language recognition, various researchers have proposed systems with high recognition rates. Sign language recognition (SLR) has made significant progress in achieving high recognition accuracy in recent years with the development of practical deep learning architectures and rapid improvements in computing power. In sign language video recognition, a standard method is to convert video into a frame-by-frame in pre-processing, obtain position information of fingers, arms, nose, mouth, etc., by optical flow and posture estimation process for the image, and model spatiotemporal information using Three-Dimensional Convolutional Neural Network (3DCNN) [1] in the machine learning process of sign language recognition. In particular, models using posture estimation with MMpose [2], OpenPose [3], or MediaPipe [4] have been proposed and tend to have higher recognition rates [5,6,7,8,9,10,11,12,13,14,15,16,17,18,19]. Recently, there has also been an increase in re-studies that use multiple recognition methods and fuse their results to further improve the recognition rate. In particular, the fusion of individual modalities has been performed recently to further improve the recognition rate.

In this study, we focus on an isolated SLR task and use RGB video from a dataset AUTSL [20] of Turkish Sign Language. This dataset was used in the competition used in the workshop and is readily available and easy to use. The baselines used in this competition were CNN, FPM [21], BLSTM [22], and model of attention, with a recognition rate of 49.23%. Sincan et al. summarized this competition’s results [23]. The main results using the RGB dataset used in this study are shown below. The recognition rate results below also differ because the training data included training and validation data. In contrast, only training data were used in this study, with the former having higher recognition rates.

- Coster et al. proposed a model Video Transformer Network-Pose flow (VTN-PF) that provided posture information or hand geometry from RGB video data frame by frame to the Video Transform Network. They achieved a recognition rate of 92.92% [24].
- The Wenbinwuee team trained multiple models for RGB video recognition using RGB, optical flow, and person segmentation data, obtained the final prediction for each model using SlowFast, SlowOnly, and Temporal Shift Module (TSM), and fused the results, and obtained a result of 96.55% [23].
- The rhythmblue6 team proposed an ensemble framework consisting of multiple neural networks (Inflated 3D (I3D), Semantics-Guided Neural (SGN), etc.) and implemented the University of Science and Technology of China-Sign Language Recognition (USTC-SLR) model for isolated characters, 97.62% [23].
- Jiang et al. obtained 98.42% for Skeleton Aware Multi-modal-Sign Language Recognition (SAM-SLR), 97.62% for the first version [14], and 98.00% for the second version [15] when trained on training data alone.

The main results published on the website of AUTSL Benchmark (https://paperswithcode.com/sota/sign-language-recognition-on-autsl accessed on 9 April 2023) are as follows.

- Novopoltsev et al. proposed a real-time recognition system using the Video Swin transformer [25] and Multiscale Vision Transformers (MViT) [26] models. They achieved a recognition rate of 95.72% and 2–3 predictions per second on CPU [27].
- Ryumin et al. proposed audio-visual speech recognition using spatio-temporal features (STF) and long-short term memory (LSTM) models. The model is especially characterized by the incorporation of lip information. They achieved a recognition rate of 98.56% and demonstrated a real-time process using mobile devices [28].

In addition to the above, the results published in the paper are as follows.

- Hrúz et at. analyzed 2 appearance-based approaches, I3D and TimeSformer, and 1 pose-based approach, SPOTER, which achieved recognition rates of 96.37% and 97.56% for test and validation datasets, respectively [16].
- Al-Hammadi et al.’s proposed architecture consists of a few separable Three-Dimensional Graph Convolution Network (3DGCN) layers, which are enhanced by a spatial attention mechanism. They achieved a recognition rate of 93.38% [17].
- We proposed a method to reuse the estimation results produced at each epoch based on SAM-SLR, which improved the recognition rate to 98.05% [19].

This section describes the SAM-SLR model used in the proposed methodology. It should be noted that this model was also used in the previously proposed paper [19], so the description is almost identical. In RGB video sign language recognition, there are four modalities: RGB-frames, RGB-flow, features, and multi-stream, each of which independently performs sign language recognition and extracts features.

We will next talk about pre-processing, the four modalities, and late fusion.

- For pre-processing, the RGB stream uses the TVL1 algorithm [29], an OpenCV, and CUDA implementation of the Denseflow API provided by OpenMMLab [30], to extract the optical flow. The features and multi-stream modalities used MMPose [2] to extract the pose estimation.

The four modalities are RGB-frames, RGB-flow, features, and multi-stream, each of which independently performs sign language recognition and extracts features.

- The RGB-frames and RGB-flow modalities are modeled in a 3DCNN [1] using the ResNet2+1D [31] architecture, which separates the temporal and spatial convolution of a 3DCNN. The model chooses the Res-Net2+1D-18 variant for its backbone, which is pre-trained on the Kinetics dataset [32]. In addition, it is pre-trained on the most extensive available SLR dataset SLR500 [33] for the RGB frame to further improve the accuracy.
- A separate spatial–temporal convolutional network (SSTCN [14]) was developed to learn from the entire skeleton to fully extract information from key points throughout the body.
- A multi-stream sign language graph convolutional network (SL-GCN [14]) was designed to model the embedding dynamics using the whole-body key points extracted by the pre-trained whole-body posture estimator. The estimated results and weights of the joint, bone, motion joint, and motion bone streams were multiplied and used as the evaluation value of the multi-stream modality. The highest recognition rate among the modalities was achieved by multi-stream, which had a recognition rate of 96.47%.
- There are two versions of late fusion.
- The first version [14] proposes ensemble model-free late fusion, a simple late fusion approach that fuses predictions from all modalities. All modalities were manually weighted using {1.0, 0.9, 0.4, 0.4}. The recognition result was 97.62%.

In the second version of SAM-SLR [15], a learning global ensemble model was proposed because finding the optimal weights for fusion is time consuming. The method was pre-trained in a neural network with the score of each modality as input and each weighted as output. The recognition result was 98.00%. For the other datasets, the recognition rates were also high for WLASL2000 [34] after pre-training on SLR500 [33] and the BSL-1K [35] dataset (Table 1).

The AUTSL dataset used in this study already achieved a recognition rate of over 98% in 2021. Thus, datasets and models, which are already in a high recognition rate, are in a situation where the recognition rate cannot be easily improved. The test data for this dataset contain datasets that are difficult for humans to judge, so it may be difficult to answer all of them correctly. In addition, a report on the recognition rate of the validation set for this dataset showed that the percentage of samples correctly recognized by at least one model was 98.96% [16], suggesting the possibility of eventually achieving recognition rates of around 99%. We proposed a re-evaluation method to further improve the recognition rate. In 2023, before our proposal, Ryumin et al. proposed a model to reach 98.56% [28].

In our approach, we decided to re-evaluate the results from Top-1 to Top-3, noting that the recognition rate tends to be lower when the difference between the Top-1 and Top-2 scores among the recognition results of the first version of SAM-SLR is slight.

The evaluation method was based on the fact that although the positions of facial parts such as eyes, nose, and mouth differ from person to person, by creating a triangle with the facial parts as vertices and connecting the vertices, the coordinates of the index finger of the dominant hand can be converted to the coordinates inside the triangle. This method is based on the fact that the position of the finger can be captured from the position of the facial parts and compared with four locations where the finger is considered stationary.

Our main contributions can be summarized as follows. Based on the results of the SAM-SLR evaluation, we re-evaluated only the data with low recognition rates to maintain the recognition rate. The proposed recognition method, which is not used in SAM-SLR, independently reflects the recognition rate and can achieve a higher recognition rate than that of SAM-SLR. Since the information converted from the finger position information to the face part coordinates from the training data is stored in advance and only converted to face part coordinates from the test data at the time of re-recognition, the computation is reduced. We will report comparative results when the proposed method is applied to each of the first versions of SAM-SLR (hereafter referred to as SAM-SLR) and the previously proposed model. This method can be applied to machine learning in the future.

## 2. Methodology

### 2.1. Dataset

The AUTSL [20] dataset was created for sign language recognition competitions and contains Turkish Sign Language with 36,302 video files each of RGB and depth video (Table 2). This dataset is publicly available on the Internet and can be easily obtained by anyone. The videos are all 512 × 512 in size, with the signer standing or sitting almost in the center. Only RGB video is used.

### 2.2. Architecture of SAM-SLR

**Figure 1:**
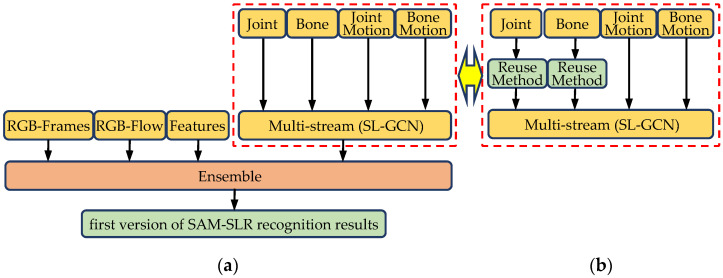
Concept of skeleton aware multi-modal sign language recognition framework (SAM-SLR) (RGB version). (**a**) Original [14,15] and (**b**) Last ours, our proposed joint and bone streams in the multi-stream modality [19].

SAM-SLR uses four methods from the sign language videos, including optical flow, 3DCNN, and posture estimation, and synthesizes the results of each estimation to achieve a high recognition rate. On the Turkish sign language dataset, the first version achieves a recognition rate of 97.62%, and the second version achieves a recognition rate of 98.00%. In addition, the method we proposed in the previous study (Figure 1), which reuses the estimation results of the training models created in each of the previous epochs of joint and bone, 2 streams used in the multi-stream modality, and one of the modalities of SAM-SLR, improved the recognition rate to 98.05% [19]. However, a higher recognition rate could not be achieved. Therefore, a new recognition method is needed to seek a higher recognition rate, and we have three perspectives on the new recognition method. The first perspective is a method to re-evaluate the 2% portion that is not recognized from the recognition results of the first version of SAM-SLR (hereafter referred to as SAM-SLR). The second perspective was that some characters touching the mouth, eyes, nose, etc., could not be recognized because the position of the fingers could not be accurately detected. The third perspective detects those index fingers that stay longer near each facial part from the training information in the dataset, captures their relative position, creates a heatmap for each sign language, and compares it with the relative position of the index finger in the test data for recognition processing.

### 2.3. Overview of the Re-Evaluation Method

**Figure 2:**
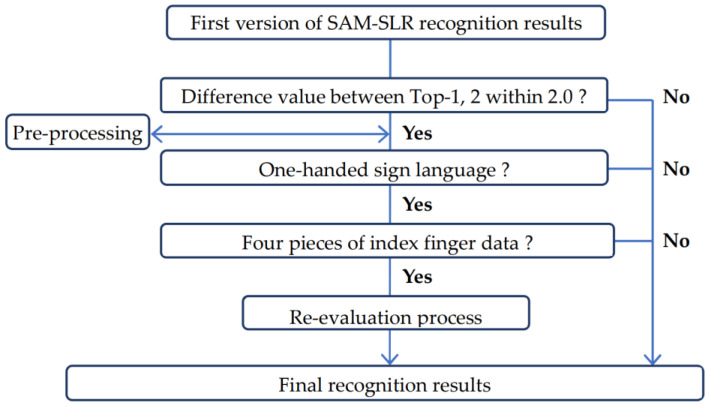
Flowchart of the re-evaluation process. Example: difference value is within 2.0, 1-handed sign language and 4 pieces of finger data.

The flowchart diagram (Figure 2) illustrates the overall process of the re-evaluation method. First, as a pre-processing step, all sign language video data in the dataset are automatically flipped and aligned so that the dominant hand is on the right. Next, the input data are re-evaluated if they meet 3 conditions (the difference between the Top-1 and Top-2 evaluation values from the SAM-SLR recognition results is within 2.0, the sign language is 1-handed, and there are 4 index finger position data in the face area of the test data). Finally, the results of this evaluation are replaced with the SAM-SLR results. This set of procedures was automatically determined by the amount of movement of each left and right finger when judging one-handed signs, and any outlier information in the posture estimation was excluded from obtaining the correct index finger position information.

#### 2.3.1. Difference Value between Top-1 and Top-2

For the first method, Table 3 shows the difference between the Top-1 and Top-2 scores and the percentage of correct responses for the test data of the training model at epoch 196 of the SAM-SLR. Note that the maximum value of this difference was up to 14.0. This table shows that a low percentage of correct answers characterizes those with a slight difference between the Top-1 and Top-2 scores. This is because the sign languages in these classes are considered to be similar and difficult to distinguish. If some recognition methods from a different perspective than the one used in SAM-SLR could be used, the current recognition rate could be exceeded. In particular, with a difference of 2.0 in the evaluation value, the recognition rate is 68.94% (Table 3). Hypothetically, if we could achieve a recognition rate of about 75% with a new method in the 132 tests data, we would be able to increase the number of correct answers in the test data by about 6%, which would increase the number of correct answers by about 8 data equivalents. As a result, the number of correct answers in the SAM-SLR would increase from 3665 to 3673, and the overall recognition rate would reach 98.16%.

Conversely, suppose the recognition rate of this method is about 75%. In this case, the recognition rate for a difference of 3.0 in the evaluation value is as high as 75.66%, so it is expected that the recognition rate will decrease when using this method, and the more significant the difference in the evaluation value, the lower the recognition rate. Therefore, we will consider the case where the difference in the evaluation values is 2.0.

Regarding the range of classes to be re-evaluated, the correct answers for Top-1, Top-2, and Top-3 were 3665, 3727, and 3739, respectively. The recognition rates were 97.94%, 99.60%, and 99.92%, respectively, and since the range up to Top-3 can almost cover the whole range, the scope of the re-evaluation was limited to Top-3.

#### 2.3.2. One-Handed or One- or Two-Handed Sign Language

The default for the second conditional branch in the flowchart was set to one-handed sign language. The two reasons for this are that the re-evaluation method is aimed at sign language that touches parts of the face area using the index finger, many of which require one-handed sign language, and to improve the recognition rate. In Chapter 3, we examine one-handed or one- or two-handed sign language cases.

#### 2.3.3. Number of Index Finger Data

The default for the last conditional branch in the flowchart is 4 samples of index finger position information. That is because a small sample size will include sign language in which the index finger just passes through the face area while setting a larger sample size will reduce the number of test data that meet the criteria. In Chapter 3, the number of samples of this index finger is verified from 2 to 7.

### 2.4. Index Finger Position in Face Area Using Face Part Position Criterion

**Figure 3:**
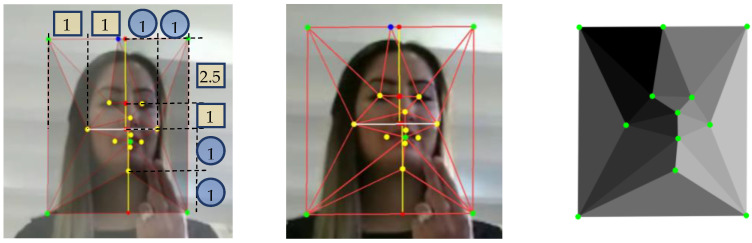
Structure of face triangle mesh from the AUTSL [20] dataset.

The second method is to touch parts of the face with the index finger. OpenPose [3] is used for posture estimation. The position of the parts of the face varies from person to person, such as the size of the face or the elongation. In order to absorb this difference in position, a skeleton (Figure 3) composed of triangles is created with each facial part as a vertex, and the number of the triangle in which the index finger is located and the position of the index finger within that triangle are stored. This allows for more accurate position comparisons. To capture the relative facial structure, we used the positional information of the face’s eyes, nose, mouth, cheeks, and chin used in SAM-SLR (yellow dots) to create a new vertex (green dots) to fit the contour of the face. The upper vertex was stretched to 2.5 times its height when the height from the midpoint of the cheek edges and eyes on each side to the intersection with the perpendicular line was set to 1. The lower vertex was further stretched to the same height when the height to the intersection of the cheek edges and the perpendicular line from the chin was set to 1. The outer left and right vertex positions were created from the left and right cheek vertexes and the chin vertex, with their lower vertexes twice as high as the diagonals perpendicular to each intersection of the cheek edges and the chin. To capture the relative position of the fingers, a mesh of 17 triangles was created using these vertices.

**Figure 4:**
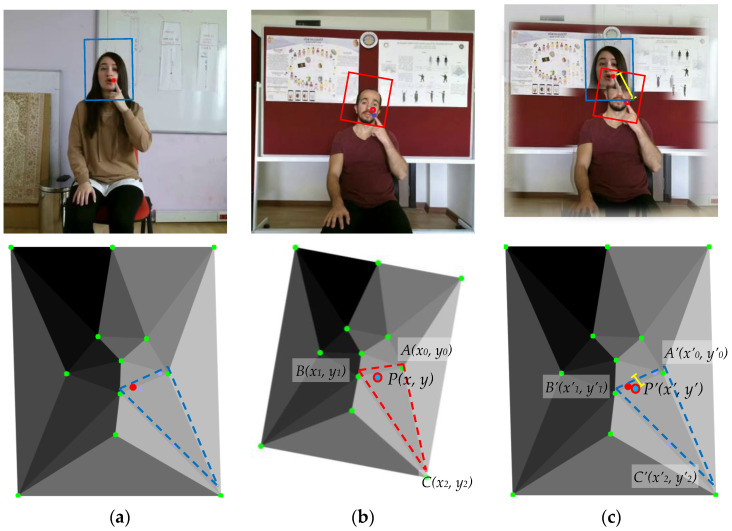
Improvement of index finger position in face area using face part position criterion from the AUTSL [20] dataset: (**a**) test data, (**b**) training data, and (**c**) distance differences of the positioning of the index finger.

Figure 4 shows the sign language with the index finger pointing to the mouth, but in the top row, the positioning of the index finger is very different. In the lower row, the coordinates of the index finger are recorded on the triangle mesh based on the facial parts, and when the coordinates within the triangles (red line) of the trajectory data (b) are transformed affine to the coordinates within the triangles (blue line) of (a) and (c), the positions of each index finger are very close.

### 2.5. Process of Staying Fingertip Decision

We will now discuss the decision about the object to be recognized using the new method. As mentioned above, the target should have a slight difference between the Top-1 and Top-2 values, be a one-handed sign language, have no outliers, and have all four pieces of finger information in the face area. One-handed sign language is used for signs expressed by touching parts of the face with the index finger. The reason for the "no outliers" mentioned above is that if there are outliers, the structure of the triangular network will be significantly disrupted when it is created. The reason for the outliers is that the position of a hand in the face area cannot be detected accurately because the hand hides the face. In addition, the method that uses the positions of the cheeks, chin, and eyes to capture the facial structure cannot process the face correctly because of the collapse of the facial contours.

In this study, we examined the index finger within the face region to capture the state of the index finger within the face region, which is the smallest distance the index finger moves within the face region. The method was to sum the distance traveled between three frames, one before and one after each frame, and those with the shortest distance traveled were judged to have stayed longer in that location. We decided to extract the Top-4 shortest travel distances in the order of shortest travel distance. Fingers in the three frames before and after the adopted *t*, i.e., seven frames, were judged to belong to the location at the adopted *t* and excluded from subsequent adoption decisions.

(1) $$d_{i}=\sum_{t=i-1}^{i=2}\sqrt{{\left(x_{t+1}-x_{t}\right)}^{2}+{\left(y_{t+1}-y_{t}\right)}^{2}}$$

**Figure 5:**
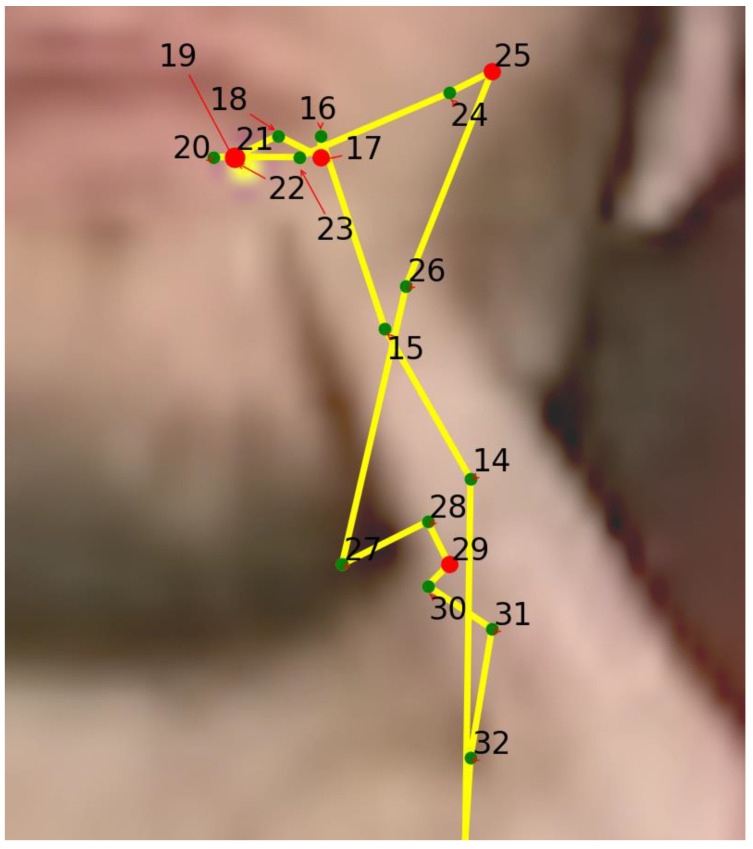
Example of moving fingertip from the AUTSL [20] dataset. (Each number at the fingertip in the figure is the frame number.)

Figure 5 shows the motion of the index finger, with the red dot indicating the adopted location. The Table 4 on the right shows the actual position information of the index finger and the total distance traveled. The yellow cells show the immediately before the adopted area that cannot be adopted again, the red letters show the actual adopted location, and the light blue cells show the second candidate area. The second possible candidate location is the light blue area from *t* = 14...17, 25...32, where *t* = 18...24 is excluded because 21 was adopted just before. The shortest, *t* = 17, was accepted at 3.2361. The last matches are 21, 17, 29, and 25.

### 2.6. Re-Evaluation Process

The Top-4 coordinates at which the index finger remained within the face region are recorded for training and test data. An affine transformation matrix was obtained to transform the coordinates of the training data collected for each class into the coordinates corresponding to the Top-1 face part coordinates of the test data. The variables in the following equation are the coordinates *A*(*x*_0_, *y*_0_), *B*(*x*_1_, *y*_1_), and *C*(*x*_2_, *y*_2_) of the triangles corresponding to the coordinates of the training data, and the triangles of the test data and their coordinates *A*’(*x’*_0_, *y’*_0_), *B*’(*x’*_1_, *y’*_1_) and *C*’(*x’*_2_, *y’*_2_) corresponding to the triangles of the training data.

(2) $$\left(\begin{array}{ccc} x_{0}^{’} & x_{1}^{’} & x_{2}^{’}\\ y_{0}^{’} & y_{1}^{’} & y_{2}^{’}\\ 1 & 1 & 1 \end{array}\right)\;=\;\;\left(\begin{array}{ccc} a & b & c\\ d & e & f\\ 0 & 0 & 1 \end{array}\right)\left(\begin{array}{ccc} x_{0}^{} & x_{1}^{} & x_{2}^{}\\ y_{0}^{} & y_{1}^{} & y_{2}^{}\\ 1 & 1 & 1 \end{array}\right)$$

The equation for finding the coordinates using the affine transform matrix:

(3) $$\hat{x}=ax\;+by\;+c\;\hat{y}=dx\;+ey\;+f$$

To create the histogram data, the image area of $\hat{x}$ and $\hat{y}$ was 480 in height and width, and the bin was 300 in the histogram.

(4) $$X=\frac{\mathrm{width}}{\mathrm{bins}}\hat{x},\;Y=\frac{\mathrm{height}}{\mathrm{bins}}\hat{y}$$

Histogram equation:

(5) $$Histogram(X,\;Y)\;=\frac{1}{n}\sum_{i=0}^{fingers(\hat{x},\;\hat{y})}H(X,\;Y)$$

After creating a histogram using the data H(X, Y) transformed for the histogram, the data were divided by the amount of data n after tabulation to equalize each class’s different amounts of data.

Gaussian filter equation:

(6) $$g(x,\;y)\;=\frac{1}{2\pi \sigma^{2}}e^{-(\;x^{2}+y^{2}\;)/(\;{2\sigma }^{2}\;)}$$

Heatmap equation:

(7) Heatmap(X, Y)=Histogram(X, Y)· g(X, Y)

**Figure 6:**
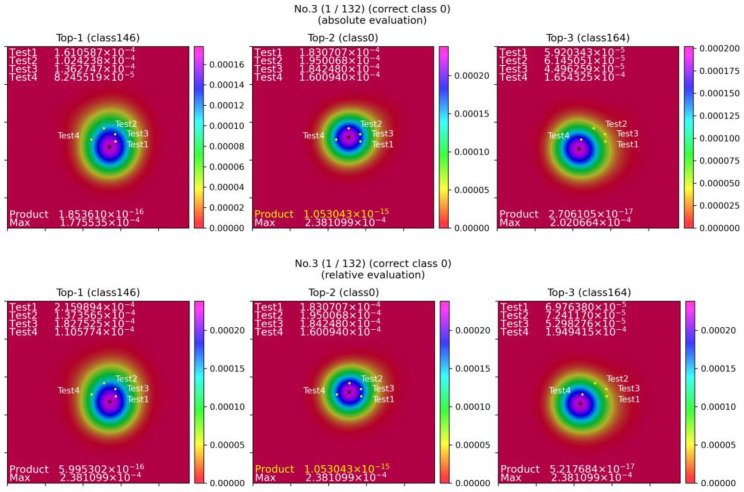
Example of absolute and relative evaluation heatmaps.

There are two types of heatmaps: absolute scores (Figure 6, top) and relative scores (Figure 6, bottom).

The heatmap uses this equation, and sigma applied a Gaussian filter of 24.

The absolute product equation:

(8) $${Absolute\;Product}_{Top-n}={\Pi Heatmap(X,\;Y)}_{Top-n}$$

For the absolute rating heatmap, we created a heatmap of the top 1, 2, and 3 histogram data for each class candidate. We obtained the product of the heatmap values at the four coordinates of the test data.

The relative product equation:

(9) $${High\;Absolute\;Product}_{Top-n}=Max\left({\Pi Heatmap(X,\;Y)}_{Top-n}\right)\;{rate}_{Top-n}=\frac{Max\left({High\;Absolute\;Product}_{Top-1,\;2,\;3}\right)}{{High\;Absolute\;Product}_{Top-n}}\;Relative\;{Product}_{Top-n}={\Pi Heatmap(x,\;y)}_{Top-n}\cdot {rate}_{Top-n}$$

The relative rating value heatmap was created by multiplying the highest absolute rating value in the Top-1, 2, and 3 classes by the highest value in each class so that the highest value in each class is the same. We obtained the product of the heatmap values at the four coordinates of the test data. The plotted positions of Test1 to Test4 are the positions of the index fingers of the test data in order of most extended stay, and each value is the heatmap value of the training data at that position.

There are three scoring methods: The first and second are based on the product of the values of the four finger positions of the test data in the heatmap of the finger positions of the training data made for each class, using the heatmap of absolute evaluation values for the former and the heatmap of relative evaluation values for the latter. The third method is an evaluation method that integrates these two heatmaps, where the first place is given three points, the second place two points, and the third place one point, and each class is added up, and if there is a tie for the first place, the class with the best relative evaluation value is selected.

Figure 6 shows the heatmap output for Top-1 to Top-3, where the correct answer for a given test data is class 0. From left to right, the SAM-SLR recognition results show that Top-1 is class 146, Top-2 is class 0, and Top-3 is class 164. The absolute evaluation of the top row shows that the product of Top-2 has the highest value, and Top-2 is selected as the re-evaluation result. The black dots in the heatmap’s center indicate the maximum value’s location. In the relative evaluation at the bottom, the highest value of the maximum relative evaluation for each class is 2.381099 × 10^−4^, which is the maximum value of Top-2, so each rate in Equation (7) is multiplied on each heatmap so that the maximum value for each class is this value. This results in the values of Top-1 and Top-3 being 5.995302 × 10^−16^ and 5.217684 × 10^−17^, respectively, and the value of the product of Top-2 being 1.053043 × 10^−15^, which is the same as the value of the absolute score. Since this value is the highest, Top-2 is selected as the re-evaluation result.

## 3. Results

In this section, we present the results of re-evaluation experiments based on the models (1) SAM-SLR and (2) SAM-SLR with the last our model.

### 3.1. PC Environments

We used the published code used in SAM-SLR [15], created training models for each method on our PC, used test data, and produced estimated results. The development environment and evaluation methods are based on the same criteria as the previously proposed paper [19]. Our PC specs are AMD 3960x (CPU), RTX-3090 (3 GPUs), 128 GB (RAM), AMD 3950x (CPU), RTX-2080Ti (GPU), 64GB (RAM), and Ubuntu 18.04 (OS).

### 3.2. Summary of Results

The test data consist of 3742 RGB videos, each video containing 1 of 226 different signs. The evaluation method outputs an estimation result for 226 different signs per video. For the evaluation method of recognition rate, the number of correct answers is counted as Top-1 when the correct answer is the highest in the evaluation value for that class.

(10) $$Recognition\;rate\;of\;To\mathrm{p-1}=\frac{Total\;number\;of\;To\mathrm{p-1}}{3742}$$

Each parameter of the heatmap used in this study has a histogram interval of 300 and a heatmap sigma of 24. There are four scoring models: SAM-SLR, the previously proposed model (Last ours), and Ours-1 and Ours-2, which add the method proposed here to these two models. The recognition rates were 97.94%, 98.05%, 98.24%, and 98.21%, respectively (Table 5). Last our model is a method to improve the recognition rate of SAM-SLR by reusing the evaluation values of the training models generated at each epoch of the joint and bone streams, which are part of the multi-stream model using the SAM-SLR posture estimation. When the data and the PC used were run in the same environment, including SAM-SLR, the recognition rate of the proposed method Ours-1 showed the highest value, 0.3 points higher than SAM-SLR, and 0.21 points higher than the second version of SAM-SLR in the paper. 

**Figure 7:**
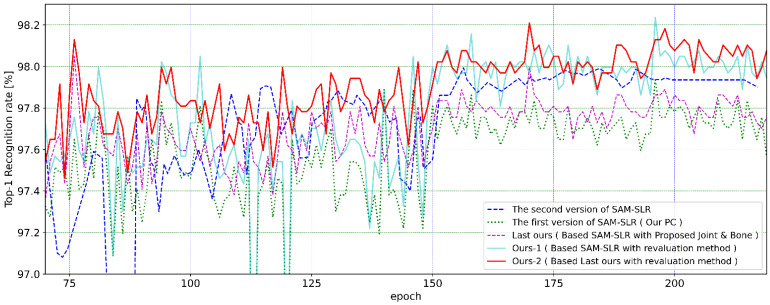
Performance of our re-evaluation results from each epoch evaluated by models on the AUTSL test set. (The second version of SAM-SLR, the first version of SAM-SLR, and Last ours [19]).

The following table (Table 6) shows the results of the recognition rates for absolute, relative, and combined absolute and relative ratings and the average recognition rate at epochs 150 to 229 using the 2 models, Ours-1 and Ours-2. The order of the highest recognition rates was higher for the integrated, relative, and absolute scores, and the model was slightly higher for Ours-1. For the integrated score, the results were 98.24% and 98.21% for each model, respectively. In that order, the average recognition rate was also higher for the integrated, relative, and absolute scores, with a slightly higher rate for the Ours-2 model. For the integrated score, the results were 98.00% and 98.04% for each model, respectively. Compared with the results of the second version of SAM-SLR, created visually from the paper but only as a reference due to different PC environments, the last proposed method (Last ours) achieved 98.05%. However, the average recognition rate of the second version compared with the graph was better. On the other hand, due to improvements, Ours-1 achieved the highest recognition rate of 98.24% and Ours-2 had the highest average recognition rate of 98.04%. Moreover, focusing on the point of concern in this graph, there are times when the recognition rates of SAM-SLR and Ours-1 drop significantly, about 6 times between epochs 80 and 150, but there are also times when the model using posture estimation drops significantly at the same time, which could be the cause. Applying the previous model improved this significant drop, and the Ours-2 with the Last our model applied remained stable and high. In the graph, the recognition rate improves dramatically from epoch 150. This is because the posture estimation model used in SAM-SLR is set to suppress the learning rate to one-tenth of the standard rate when learning from epochs 150 to 200, contributing to the improved recognition rate. Therefore, we started evaluating the average recognition rate at epoch 150 (Figure 7).

### 3.3. The Performance of Each Conditional Branch

The recognition rate before introducing this method was 97.94%, and after the introduction, the recognition rate improved except the rate for conditional branches on 1 or 2-handed sign language and 3 pieces of index finger data was 97.92%. The average recognition rate for these cases was 98.09%, an improvement of 0.15 percentage points. In addition, the maximum recognition rate was 98.24% for 1-handed sign language, with 4 pieces of index finger dataset as the default for the conditional branch.

For the first conditional branch, regarding the difference value between Top-1 and Top-2, we predicted in the previous section that a difference of 2.0 would be optimal. The average recognition rate before the introduction was 69.94%, but from Table 7, it can be observed that the average recognition rate for the entire test data after the re-evaluation was 72.98% and the high recognition rate was 77.27%.

For the second conditional branch, one-handed sign language, the recognition rate improves when introduced from the beginning since many sign language signs that touch parts of the face area using the index finger are one-handed sign language signs. In the case of 1-handed sign language, the average was 98.12%, while in the case of 1 or 2-handed sign language, i.e., without this decision, the average was 98.05%. In addition, the number of re-evaluated cases averaged 30.0 for 1-handed sign language, while it was 35.8 for 1 or 2-handed sign language, with the latter having 10 more cases.

The third conditional branch, the number of index finger data, was higher, with an average of 98.20% when there were 4 samples and 97.99% when there were 3 samples. When looking at the re-evaluated cases, the number of cases and their recognition rate averaged 47.5 cases and 68.42% when there were 2 samples. At the same time, 13.5 cases and 88.89% were recognized when there were 7 samples. When the number of samples was small, the number of cases increased, and the recognition rate was low, while when the number of samples was large, the number of cases decreased, and the recognition rate tended to be high.

### 3.4. The Performance of Each Epoch

**Figure 8:**
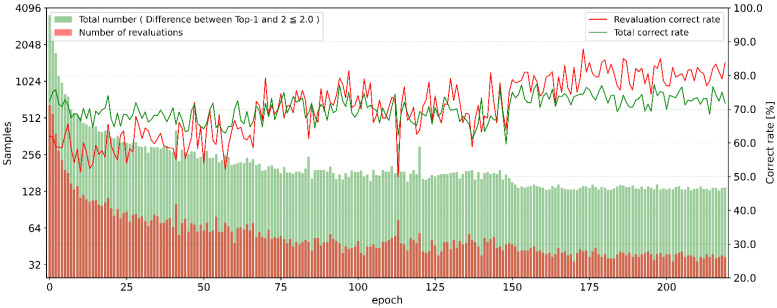
The difference between the Top-1 and Top-2 evaluation values is within 2.0, the number of the re-evaluation and the percentage of correct answers for each.

The graph in Figure 8 shows the cases where the difference between the Top-1 and Top-2 evaluation values is within 2.0 using the Ours-1 method, the number of cases retested, and the percentage of correct answers for each. The number of cases within 2.0 and the number of cases resurveyed at epoch 0 were 3557 and 651, respectively, and the number gradually decreased, averaging 136.8 and 39.1, respectively, from epoch 150 to epoch 229. The percentage of resurveyed cases averaged 26.87% for epochs 0 to 229. Although some cases were excluded for outliers or other reasons, the percentage of signs touching the face in 1-handed sign language is just under 30%. Regarding the percentage of correct answers in the resurvey and the percentage of correct answers within 2.0, the averages from epochs 150 to 229 were 79.8% and 73.3%, respectively. Within 2.0 was the best for this method since a difference in scores greater than 2.0 could result in a lower recognition rate. In addition, since about 70% of the areas were still not re-examined, the recognition rate could be improved in the same way by trying another analysis method using the re-evaluation method proposed in this study.

## 4. Conclusions

In the previous method, we proposed a method to reuse the results of the recognition estimation of the training model, which reached 98.05%. Compared with the SAM-SLR, the results were generally good in the recognition rate of each epoch after epoch 150. However, there were few cases exceeding 98%; therefore, in this study, we proposed a method to accurately capture the location of the index finger in the face region to improve the recognition rate. As a result, we found that the difference between Top-1 and Top-2 of the SAM-SLR estimation results was within 2.0. In the method of re-evaluation when four location data of the stopped finger could be obtained, the recognition rates of the SAM-SLR-based method and the previously proposed method were, respectively, 98.24% and 98.21%, with the former being better and improving by up to 0.3, 0.24, and 0.19 percentage points, respectively, when compared with the first version and the second version of SAM-SLR, and the previous method. The average recognition rate from epochs 150 to 229 was 97.79% for the previously proposed method and 98.00% and 98.04 for the proposed former and latter, respectively, improving by 0.21 and 0.25 points, respectively. Since this method was relatively independent of the SAM-SLR recognition methods, it is thought that it could reflect the recognition results without being affected by other modalities.

There are three points to keep in mind when applying the re-evaluation method to other models.

- The recognition rate with slight differences in top-1 and top-2 evaluation values in the SAM-SLR recognition results is low (e.g., 75% or less).
- The recognition rate up to Top-3 is close to 100%.
- In the face area, no special recognition processing was performed.

Our re-evaluation method can process the re-evaluation by an average of 0.101 s per video using CPU when setting each conditional branch is the default after the SAM-SLR recognition results process.

## Figures and Tables

**Table 1:** Summary of isolated sign language datasets.

Datasets	Language	Glosses	Signers	Samples
AUTSL [20]	Turkish	226	43	36,302
SLR500 [33]	Chinese	500	50	125,000
WLASL2000 [34]	American	2000	119	21,083
BSL-1K [35]	British	1064	40	273,000

**Table 2:** A statistical summary of the AUTSL [20] Dataset ([19]).

Dataset	Signs	Signers	Language	Frames	Samples			
					Training	Validation	Testing	Total
AUTSL	226	43	Turkish	57–157	28,142	4418	3742	36,302

**Table 3:** Value of difference between Top-1 and Top-2 on the first version of SAM-SLR results.

Value of Difference	Number of Matched	Number of Correct	Number of Incorrect	Top-1 Acc (%)
1.0	64	38	26	59.38
2.0	132	91	41	68.94
3.0	226	171	55	75.66
…				
9.0	1984	1907	77	96.12
…				
14.0	3742	3665	77	97.94

**Table 4:** Process of staying fingertip decision.

*t*	*x*	*y*	*d_i_*	1st	2nd	3rd	4th
14	266	130	13.0064		−3		
15	262	123	17.5491		−2		
16	259	114	10.4868		−1		
17	259	115	3.2361		0		
18	257	114	4.4721	−3	1		
19	255	115	3.2361	−2	2		
20	254	115	2.0000	−1	3		
21	255	115	1.0000	0			
22	255	115	3.0000	1			-3
23	258	115	10.6158	2			-2
24	265	112	9.8518	3			-1
25	267	111	13.0064				0
26	263	121	24.1120			−3	1
27	260	134	17.8138			−2	2
28	264	132	6.7082			−1	3
29	265	134	3.6503			0	
30	264	135	5.0198			1	
31	267	137	9.6883			2	
32	266	143	53.1784			3	

**Table 5:** Performance of our re-evaluation results (with and without fine-tuning using the validation set) evaluated on the AUTSL test set (Baseline, VTN-PF and SAM-SLR [19]).

Model	Fine-Tune	Publication	Our PC
Baseline [20]	-	49.22	-
VTN-PF [24]	With validation data	92.92	-
Enhanced 3DGCN [17]	No	93.38	-
MViT-SLR [27]	-	95.72	-
Wenbinwuee team [23]	With validation data	96.55	-
Neural Ens. [16]	No	96.37	-
USTC-SLR [23]	With validation data	97.62	-
Second version of SAM-SLR [15]	No	98.00	-
STF + LSTM [28]	-	98.56	-
SAM-SLR [14]	No	97.62	97.94
+Last ours (proposed joint and bone streams) [19]	No	-	98.05
+Ours-1 (re-evaluation method)	No	-	98.24
+Ours-2 (Last ours and re-evaluation method)	No	-	98.21

**Table 6:** Performance of our re-evaluation results evaluated by the evaluation method on the AUTSL test set.

Based Model	Re-Evaluation Method (Finger Position Evaluation)	Acc.	Average of Acc. From Epoch 150 to 229
SAM-SLR [14] (Ours-1)	-	97.94	97.73
	Absolute	98.05	97.82
	Relative	98.16	97.92
	Absolute and relative	98.24	98.00
SAM-SLR with proposed joint and bone streams [19] (Ours-2)	-	98.05	97.79
	Absolute	98.00	97.82
	Relative	98.13	97.96
	Absolute and relative	98.21	98.04

**Table 7:** Performance of absolute and relative re-evaluation results evaluated depending on one-handed sign language or one- or two-handed sign language and the number of finger data by the evaluation method on the AUTSL test set.

	Number of Index Finger Data					
	2	3	4	5	6	7
	One-handed sign language					
Total number	132	132	132	132	132	132
Number of correct	98	95	102	99	97	95
Correct rate (%)	74.24	71.97	77.27	75.00	73.48	71.97
Number of re-evaluations	41	41	35	30	20	13
Number of correct	30	27	29	22	17	12
Correct rate (%)	73.17	65.86	82.86	73.33	85.00	92.31
Final recognition rate (%)	98.13	98.05	98.24	98.16	98.10	98.05
	One- or two-handed sign language					
Total number	132	132	132	132	132	132
Number of correct	93	90	99	98	96	94
Correct rate (%)	70.45	68.18	75.00	74.24	72.72	71.21
Number of re-evaluations	54	51	42	33	21	14
Number of correct	35	30	32	23	17	12
Correct rate (%)	64.81	58.82	76.19	69.70	80.95	85.71
Final recognition rate (%)	98.00	97.92	98.16	98.13	98.08	98.02

## Data Availability

This study used the following publicly available dataset: AUTSL- http://cvml.ankara.edu.tr/datasets, accessed on 17 March 2023.

## References

[1] Ji S., Xu W., Yang M., Yu K. (2013). 3D Convolutional Neural Networks for Human Action Recognition. IEEE Trans. Pattern Anal. Mach. Intell. TPAMI.

[2] Contributors M. (2020). OpenMMLab Pose Estimation Toolbox and Benchmark. https://github.com/open-mmlab/mmpose.

[3] Cao Z., Hidalgo G., Simon T., Wei S.E., Sheikh Y. (2019). OpenPose: Realtime multi-person 2D pose estimation using part affinity fields. IEEE Trans. Pattern Anal. Mach. Intell..

[4] Google Research Team (2020). MediaPipe. https://google.github.io/mediapipe/solutions/hands.html.

[5] Wang H., Wang L. Modeling temporal dynamics and spatial configurations of actions using two-stream recurrent neural networks. Proceedings of the IEEE Conference on Computer Vision and Pattern Recognition.

[6] Yan S., Xiong Y., Lin D. Spatial temporal graph convolutional networks for skeleton-based action recognition. Proceedings of the 32nd AAAI Conference on Artificial Intelligence.

[7] Shi L., Zhang Y., Cheng J., Lu H. (2020). Skeleton-based action recognition with multi-stream adaptive graph convolutional networks. IEEE Trans. Image Process..

[8] Cheng K., Zhang Y., Cao C., Shi L., Cheng J., Lu H. (2020). Decoupling GCN with DropGraph Module for Skeleton-Based Action Recognition. Proceedings of the European Conference on Computer Vision (ECCV).

[9] Jin S., Xu L., Xu J., Wang C., Liu W., Qian C., Ouyang W., Luo P. Whole-body human pose estimation in the wild. Proceedings of the European Conference on Computer Vision (ECCV 2020).

[10] Xiao Q., Qin M., Yin Y. (2020). Skeleton-based Chinese sign language recognition and generation for bidirectional communication between deaf and hearing people. Neural Netw..

[11] Song Y.F., Zhang Z., Shan C., Wang L. (2020). Stronger, Faster and More Explainable: A Graph Convolutional Baseline for Skeleton-Based Action Recognition. Proceedings of the 28th ACM International Conference on Multimedia (ACMMM).

[12] Liu Z., Zhang H., Chen Z., Wang Z., Ouyang W. Disentangling and Unifying Graph Convolutions for Skeleton-Based Action Recognition. Proceedings of the IEEE/CVF Conference on Computer Vision and Pattern Recognition.

[13] Vázquez-Enríquez M., Alba-Castro J.L., Fernández L.D., Banga E.R. Isolated Sign Language Recognition with Multi-Scale Spatial-Temporal Graph Convolutional Networks. Proceedings of the 2021 IEEE/CVF Conference on Computer Vision and Pattern Recognition Workshops (CVPRW).

[14] Jiang S., Sun B., Wang L., Bai Y., Li K., Fu Y. Skeleton aware multi-modal sign language recognition. Proceedings of the IEEE/CVF Conference on Computer Vision and Pattern Recognition.

[15] Jiang S., Sun B., Wang L., Bai Y., Li K., Fu Y. (2021). Sign Language Recognition via Skeleton-Aware Multi-Model Ensemble. arXiv.

[16] Hrúz M., Gruber I., Kanis J., Boháček M., Hlaváč M., Krňoul Z. (2022). One Model is Not Enough: Ensembles for Isolated Sign Language Recognition. Sensors.

[17] Al-Hammadi M., Bencherif M.A., Alsulaiman M., Muhammad G., Mekhtiche M.A., Abdul W., Alohali Y.A., Alrayes T.S., Mathkour H., Faisal M. (2022). Spatial Attention-Based 3D Graph Convolutional Neural Network for Sign Language Recognition. Sensors.

[18] Dafnis K.M., Chroni E., Neidle C., Metaxas D.N. Bidirectional Skeleton-Based Isolated Sign Recognition using Graph Convolution Networks. Proceedings of the 13th Conference on Language Resources and Evaluation (LREC).

[19] Hori N., Yamamoto M. Sign Language Recognition using the reuse of estimate results by each epoch. Proceedings of the 7th International Conference on Frontiers of Signal Processing (ICFSP).

[20] Sincan O.M., Keles H.Y. (2020). AUTSL: A Large Scale Multi-Modal Turkish Sign Language Dataset and Baseline Methods. IEEE Access.

[21] Sincan O.M., Tur A.O., Keles H.Y. Isolated sign language recognition with multi-scale features using lstm. Proceedings of the 27th Signal Processing and Communications Applications Conference (SIU).

[22] Graves A., Schmidhuber J. (2005). Framewise phoneme classification with bidirectional LSTM and other neural network architectures. Neural Netw..

[23] Sincan O.M., Jacques Junior J.C.S., Escalera S., Keles H.Y. Chalearn LAP large scale signer independent isolated sign language recognition challenge: Design, results and future research. Proceedings of the IEEE/CVF Conference on Computer Vision and Pattern Recognition Workshops.

[24] Coster M.D., Herreweghe M.V., Dambre J. Isolated Sign Recognition from RGB Video using Pose Flow and Self-Attention. Proceedings of the IEEE/CVF Conference on Computer Vision and Pattern Recognition.

[25] Liu Z., Ning J., Cao Y., Wei Y., Zhang Z., Lin S., Hu H. Video swin transformer. Proceedings of the IEEE/CVF Conference on Computer Vision and Pattern Recognition (CVPR).

[26] Fan H., Xiong B., Mangalam K., Li Y., Yan Z., Malik J., Feichtenhofer C. Multiscale vision transformers. Proceedings of the IEEE/CVF International Conference on Computer Vision (ICCV).

[27] Novopoltsev M., Verkhovtsev L., Murtazin R., Milevich D., Zemtsova I. (2023). Fine-tuning of sign language recognition models: A technical report. arXiv.

[28] Ryumin D., Ivanko D., Ryumina E. (2023). Audio-Visual Speech and Gesture Recognition by Sensors of Mobile Devices. Sensors.

[29] Zach C., Pock T., Bischof H. (2007). A Duality Based Approach for Realtime TV-L1 Optical Flow. Pattern Pattern Recognition, Proceedings of the 29th DAGM Symposium, Heidelberg, Germany, 12–14 September 2007.

[30] Wang S., Li Z., Zhao Y., Xiong Y., Wang L., Lin D. (2020). Denseflow. https://github.com/open-mmlab/denseflow.

[31] Tran D., Wang H., Torresani L., Ray J., Lecun Y., Paluri M. A Closer Look at Spatiotemporal Convolutions for Action Recognition. Proceedings of the IEEE Computer Vision and Pattern Recognition.

[32] Carreira J., Noland E., Banki-Horvath A., Hillier C., Zisserman A. (2018). A short note about kinetics-600. arXiv.

[33] Zhang J., Zhou W., Xie C., Pu J., Li H. Chinese sign language recognition with adaptive HMM. Proceedings of the 2016 IEEE International Conference on Multimedia and Expo (ICME).

[34] Li D., Rodriguez C., Yu X., Li H. Word-level deep sign language recognition from video: A new large-scale dataset and methods comparison. Proceedings of the IEEE/CVF Winter Conference on Applications of Computer Vision.

[35] Albanie S., Varol G., Momeni L., Afouras T., Chung J.S., Fox N., Zisserman A. BSL-1K: Scaling up co-articulated sign language recognition using mouthing cues. Proceedings of the 16th European Conference on Computer Vision (ECCV 2020).

